# Cross-sectional study of face mask use during the COVID-19 pandemic-Lusaka and Mansa Districts, Zambia, December 2020

**DOI:** 10.11604/pamj.2022.41.306.29854

**Published:** 2022-04-14

**Authors:** Ernest Kateule, Ignatius Banda, Muziya Chika, Ezekiel Tembo, Kabukabu Akufuna, Kingsley Keembe, Lorraine Chikonka, Marian Matipa Mulenga, Mitolo Musumba, Kelvin Mwakapushi, Rehab Mwanansoka, Deborah Tembo, Samantha Mwansa, Wisdom Banda, Chris Bupe, Floyd Chabu Chilufya, Given Mweene Hatyoka, Danny Kabwe, Bright Katai, Danny Mwenya Katongo, Mateyo Moyo, Misheck Mpundu, Leslie Mukamba, Maximillian Musunse, Lukundo Namukanga, Martin Nyambe Nyambe, Morgan Sakala, Judith Sakeyo, Chishiba Sepete, Charles Tembo, Richard Lubumba, Tamara Tembo, Ante Mutati, Patrick Chanda Kabwe, Nyambe Sinyange

**Affiliations:** 1 Zambia Field Epidemiology Training Program, Lusaka, Zambia,; 2 Ministry of Health, Lusaka, Zambia,; 3 Zambia National Public Health Institute, Lusaka, Zambia,; 4 Program for Advanced Malaria Outcomes, Mansa, Zambia,; 5 National Malaria Elimination Center, Lusaka, Zambia

**Keywords:** Sickle cell anaemia, micronutrients, renal function, liver function

## Abstract

**Introduction:**

coronavirus disease (COVID-19) is primarily spread through respiratory secretions of infected persons, and face mask use has shown to decrease transmission. In Zambia, anecdotal evidence indicates low face mask use among the general population. We objectively assessed face masks use among Lusaka and Mansa residents in December 2020.

**Methods:**

we conducted a cross sectional study of face mask usage in Lusaka and Mansa Districts from 16-23 December 2020. A standardized tool was used to visually observe face mask usage and correct face mask usage at various outdoor locations in Lusaka and Mansa. Logistic regression was used to determine association of face mask use and correct face mask use with selected demographic variables. Odds ratios (ORs) and 95% confidence intervals (CIs) were reported.

**Results:**

in total, 4070 persons were observed in Lusaka and 1166 Mansa Districts. Face masks usage was 24% in Lusaka and 27% in Mansa. Among the persons wearing face masks, 621 (48%) wore them correctly (52% in Lusaka and 35% in Mansa; p < 0.01 for difference). Being at a health facility (OR: 10.11 [95% CI: 7.99 - 12.81]), shopping mall (OR: 6.38 [95% CI: 5.07 - 8.03]), and school (OR: 2.39 [95% CI: 1.85 - 3.10]) were associated with wearing face masks compared to being at a bus station.

**Conclusion:**

face masks usage in public spaces was low in the two districts in Zambia, which might reduce efforts to control COVID-19. Investigating reasons for poor face masks adherence may help formulate effective strategies to increase face masks utilization in Zambia.

## Introduction

The ongoing spread of COVID-19, caused by SARS-CoV-2, remains a public health problem of international concern [1]. Since the World Health Organization´s (WHO) declaration of the virus as a pandemic in March 2020, the outbreak has had triggered substantial socio-economic pressure as several countries strive to implement response and control strategies against the rapidly growing COVID-19. Among the recommended public health measures that aim to prevent and/or control SARS-CoV-2 transmission in the community is correct and consistent wearing of face masks in public settings including; public and mass transportation, public events and gatherings, and any other place where individuals are likely to interact [1,2]. As a basic non-pharmaceutical intervention (NPI), wearing face masks is an effective means of preventing respiratory infectious diseases, which could reduce the risk of infection in the absence of a safe and effective vaccine to protect those at risk of severe COVID-19 [3-5]. However, wearing a face mask or cloth face covering in public settings is currently not accepted by individuals in some countries despite scientific evidence that face coverings help to control the spread of COVID-19 [6,7].

Although recent studies confirm the efficacy of face masks in reducing the risk of COVID-19 infection, the necessity of wearing masks by the general public during COVID-19 pandemic is under-emphasized in some countries [8,9]. Since SARS-CoV-2 can be spread by asymptomatic carriers, face coverings remain an appropriate tool in mitigating the spread of COVI9-19 [10-13]. In a systematic review by Chu *et al*. on physical distancing, face masks, and eye protection to prevent person-to-person transmission of SARS-CoV-2, found that face mask use could result in a large reduction in risk of infection [14]. Other studies also found the use of masks being strongly protective, with a risk reduction of 70% for those that always wore a mask in public settings [15,16]. A study in Hong Kong found that the odds ratio value of wearing masks in public spaces had a higher protective effective (OR = 0.36) compared to other interventions like living room disinfection (OR = 0.41) and frequent handwashing (OR = 0.58), against community spread of SARS-CoV-2 [16]. Hu *et al*. constructed a MERS determinant mathematical model and found that compared with auxiliary nursing and government publicity, wearing masks is the optimal choice for reducing the number of infections [17]. Barasheed *et al*. systematically analysed the utilization and effectiveness of masks by integrating, 12710 samples from more than 50 countries in the world, and found that wearing masks in crowded places could reduce the risk of respiratory infections by 20% [18].

Further, Aiello *et al*. carried out a randomized intervention trial in university residence halls during the influenza season and observed a 35%-51% reduction in influenza-like illness (ILI) in the mask and hand hygiene group compared with the no mask group during 2006-2007 [19]. These studies provide sufficient evidence to support experts´ recommendation on face covering in public settings such as busy shopping centres, religious buildings, restaurants, schools and public transport, where it might be hard to maintain social distance etiquette. For instance, the US CDC COVID-19 response team had recently conducted a study in 3076 U.S. countries, and found that allowing on-premises dining at restaurants was associated with 2.2 and 3.0 percentage point increases in the death growth rate 61-80 and 81-100 days, respectively, after COVID-19 restrictions were lifted (p < 0.01 for both) [20].

In Zambia, wearing of face masks in public spaces was made mandatory in June 2020 as a preventive measure to contain the spread of the COVID-19 pandemic. Like other countries in the region, Zambia has seen a spike in COVID-19 cases of the ongoing pandemic, characterized by increased severity and geographic spread [21]. Some identified challenges faced in controlling the outbreak include low risk perception and non-compliance to public health measures for COVID-19, including the use of face masks [21,22]. Face masks may offer some degree of protection and containment even in Zambia´s context; however, studies on mask use in Zambia do not exist. Wearing face masks remains an important intervention for reducing COVID-19, even as vaccine becomes available. Understanding the prevalence of face mask use and correct face mask use is important to inform public health strategies and policy. In this study, we investigated the prevalence of use of face masks and correlates among the general public during a coronavirus outbreak in two districts of Zambia.

## Methods

**Study design, sites and time frame:** we conducted a cross sectional study of face-masks usage in Lusaka and Mansa districts. To estimate the proportion of persons wearing face masks, we adopted Centers for Disease Prevention and Control´s (CDC) direct in-person observation approach recommended in health care settings to measure adherence to infection prevention and control [23]. This study was conducted as a field product assignment by the Field Epidemiology Training Program (FETP) and the selection of the study sites was determined by the presence Frontline FETP training workshops. This study was conducted in outdoor locations in two districts (one urban (Lusaka) and suburban (Mansa)) from 16 - 23 December 2020. Lusaka is the capital and largest city of Zambia with the population of about 3 million [24]. Mansa district is the provincial capital for Luapula whose main economic activities are centered on small scale trade with a large portion of the population ≈ 290,000 engaged in agriculture crop production with a small percentage involved in mining [24].

**Data collection:** we visited five different location types i.e. schools (5), shopping malls (5), health facilities (4), markets (4) and bus stations (4) in the two districts. Frontline FETP residents were trained as observers who recorded mask use for a maximum of three hours from assigned sites and noted demographic variables. Each observer was instructed to record at least 50 observations, without preference for selection at a single location. A standardized electronic tool was used to visually observe face mask and correct face mask usage at selected locations in each district. Face mask use was defined as the practice of using a face-covering device i.e. a disposable medical mask, surgical or non-surgical material (usually a cloth) affixed to an individual´s head in public settings [12]. While correct face mask use was defined as a covering of both the mouth and nose by providing a barrier to minimize the direct transmission of infective agents in public spaces [5,23]. Observers were urged to record only what they could see; for instance, visual age category (≤ 12 years = child; 13 - 19 years = teenager; ≥ 20 years = adult) and visual sex were based observers´ estimate. If a person´s face could not be properly observed, observers were instructed not to record the person.

**Variables, data management and analysis:** our outcome variables were face mask use and correct face mask use; the independent variables observed included: visual age category, visual sex, location type, and urban versus peri-urban area. The resulting data sets collected electronically using Kobo Toolbox which were extracted as XLS files, merged and check for consistency. Cleaned data set was exported into Epi Info for statistical analyses. Frequencies and percentages were calculated for mask use, correct mask use and locations. Logistic regression was used to determine statistical significance of association between independent variables and face mask use and correct face masks; the corresponding odds ratios (ORs) and 95% confidence intervals (CIs) were reported.

**Ethics approval and consent to participate:** this study was conducted as a part public health response by the Ministry of Health through Zambia National Public Health Institute´s and was granted an exemption from requiring ethics approval by Zambia National Research Authority (Reference Number NRA000011). Our investigation did not involve human subject interaction; data were collected by virtual observations without recording information on the persons observed.

## Results

A total of 5236 persons were observed; 4070 (77.7%) were in Lusaka and 1166 (22.3%) in Mansa districts. Of these, 2770 (53.1%) were females and the majority (n = 5209, 79.9%) were adults. Overall, 1303 (24.9%) persons wore face masks, face masks usage was slightly higher in Mansa (26.7%) than Lusaka district (24.4%, p = 0.16) (Table 1). Face mask use was higher in females than males (26.1% versus 23.5%, p = 0.03). Teenagers were the most common (30.8%) subgroup observed wearing face masks, followed by adults (24.1%). Overall, 51.9% of persons observed at health facilities wore face masks. Among persons observed at market spaces, only 2.4% wore face masks.

**Table 1:** demographic characteristics of face masks use and correct face masks use in Lusaka and Mansa Districts, Zambia, December 2020

Characteristics		Face masks use						Correct face masks use					
		Overall (N=5236 )		Lusaka (n=4070 )		Mansa (n=1166 )		Overall (N= 1303)		Lusaka (n=994 )		Mansa (n=309)	
		N	%	N	%	N	%	N	%	N	%	N	%
Overall		1303	24.9	994	24.4	309	26.5	621	47.7	513	51.6	108	35.0
Age	Child	42	17.3	22	15.0	20	20.8	18	42.9	10	45.5	8	40.0
	Teen	247	30.8	189	32.5	58	26.1	137	55.5	118	62.4	19	32.8
	Adult	1004	24.1	773	23.3	231	27.2	461	45.9	380	49.2	81	35.1
Sex	Male	577	23.5	429	23.0	148	24.9	301	52.2	239	55.7	62	41.9
	Female	726	26.1	565	25.6	161	28.1	320	44.1	274	48.5	46	28.6
Location	Shopping mall	482	41.0	328	49.8	154	29.8	215	44.6	170	51.8	45	29.2
	Market	27	2.4	27	2.6	0	0.0	18	66.7	18	66.7	0	0.0
	Bus stop	110	9.9	56	5.9	54	31.6	45	40.9	21	37.5	24	44.4
	Health facility	495	51.9	448	54.6	47	35.1	231	46.7	209	46.7	22	46.8
	School	189	28.5	135	22.7	54	78.3	112	59.3	95	70.4	17	31.5

Among the 1303 persons wearing face masks, 621 (47.7%) wore them correctly. The proportion of correct mask usage was higher in Lusaka than Mansa (n = 513, 51.6%; n = 108, 35.0% (p < 0.01)) (Table 1). Generally, the correct mask usage was highest (55.5%) among teenagers, however it differed in two districts (Lusaka: 62.4%; Mansa: 32.8%). More (52.2%) of males than females (44.1%) wore face masks correctly (X^2^ = 8.43, p ≤ 0.01). Correct face mask use varied by location of observation: markets (66.7%), schools/college (59.3%), health facilities (46.7%) shopping malls (44.6%) and bus stops (40.9%) (Table 1).

Being at a health facility (OR: 10.11 (95% CI: 7.99 - 12.81)), shopping mall (OR: 6.38 (95% CI: 5.07 - 8.03)), and school/college (OR: 2.39 (95% CI: 1.85 - 3.10)) were associated with wearing face masks compared to being at a bus station (Table 2). The odds of an individual wearing a mask increased significantly with sex; 1.3 times greater for females than males (p ≤ 0.01). Being at a market (OR: 2.89 (95% CI: 1.19 - 7.00)) and school or college (OR: 2.10 (95% CI: 1.30 - 3.39)) were associated with wearing face masks correctly compared to being at a bus station (Table 3).

**Table 2:** factors associated with face masks use in Lusaka and Mansa Districts, Zambia, December 2020

Characteristic	Number (Yes)	Total (Obs)	Mask use (%)	OR [95% C.I]	P-value
Sex					
Male	577	2457	23.5	-	1
Female	726	2779	26.1	1.27 (1.07-1.42)	<0.01
Age					
Child	42	243	17.3	-	1
Teenager	247	803	30.8	0.49 (0.19-1.27)	0.14
Adult	1004	4163	24.1	0.34 (0.13-0.87)	0.02
Location					
Bus stop	110	1116	9.9%	-	1
Shopping mall	482	1166	41.0%	6.38 (5.07-8.03)	<0.01
Market	27	1145	2.4%	0.22 (0.14-0.33)	<0.01
Health facility	495	954	51.2%	10.11 (7.99-12.81)	<0.01
School	189	664	28.5%	2.39 (1.85-3.10)	<0.01

**Table 3:** factors associated with correct face masks use in Lusaka and Mansa Districts, Zambia, December 2020

Characteristic	Number (Yes)	Total (Obs)	Correct Mask Use (%)	OR [95% C.I]	P-value
**Sex**					
Male	301	577	52.2	-	1
Female	320	726	44.1	0.69 (0.58-0.90)	<0.01
**Age**					
Child	18	42	42.9	-	1
Teenager	137	247	55.5	1.01 (0.20-3.62)	0.99
Adult	461	1004	45.9	0.80 (0.23-2.82)	0.73
**Location**					
Bus stop	45	110	40.9	-	1
Shopping mall	215	482	44.6	1.16 (0.76-1.77)	0.43
Market	18	27	66.7	2.89 (1.19-7.00)	0.01
Health facility	231	495	46.7	1.26 (0.83-1.92)	0.27
School	112	189	59.3	2.10 (1.30-3.39)	<0.01

## Discussion

During the COVID-19 pandemic, face masks usage in public spaces was 25% in two sampled districts of Zambia in December 2020. Face masks use was higher in females than males, although it was still low for both sexes. The odds of observing masks on individuals at a health facility were 10 times higher than that for bus stations. Being at a market and school or college were associated with wearing face masks correctly. Our study confirmed low usage of face masks despite being mandatory as a preventive measure in reducing risk of COVID-19 infection. To enhance face masks compliance among persons in public settings, there is need for effective strategies to facilitate wearing of masks. In our study, females had increased odds of wearing face masks compared to males. Similar findings were reported in a study conducted to understand the demographics of mask wearers and resistors, and the impact of mandates on mask-wearing behavior, among shoppers in Wisconsin, USA [6]. Some previous studies suggest that face masks were viewed as a sign of fragility or weakness among some men; while other studies report that women may be more likely to protect themselves and others by wearing a face mask because they handle the majority of caregiving within families [25,26]. Another reason was being aware of the pre-existing gender inequalities in social, political, and economic systems that have further been amplified by the pandemic [27]. Therefore, public health messages that focuses on aligning masks with masculinity would likely be beneficial to improve usage among males in Zambia.

We found that Lusaka residents were twice as likely to wear face-masks correctly, compared to those observed in Mansa district. Our study relates to findings that shown different mask-wearing habits between counties; counties or regions with low numbers of COVID-19 related deaths had indicated low risk perception to public health and social measures for COVID-19 despite adequate sensitisation [15,28]. In Zambia, Lusaka has borne the brunt of the pandemic, with 30% of all confirmed cases reported from Lusaka District from December 2020 to February 2021. Even though mask use was not different by district, perhaps among persons who wore face masks, those in Lusaka perceived a greater risk and were thus more likely to wear them correctly. Related accounts have indicated reasons why individuals in rural communities shun wearing masks, partly due to public health messaging that hasn´t been tailored to rural communities [29]. It is believed that the retention of health messaging is lower in rural areas than it is in urban. There are need for extra public health messaging that promotes masks in rural settings to dispel a false sense of security that rural residents are less vulnerable to COVID-19.

The WHO recommends universal masking in health facilities; defined as the requirement to wear a mask by all health workers and anyone entering the facility, no matter what activities are undertaken [1,13]. This perhaps explained why face masks usage was prominently higher among persons in health facilities. However, it was still well below full compliance at approximately 50%. Our study did not disaggregate data to provide results on health care workers (HCWs) and clients separately, this could partly influence compliance levels observed as below average rates of 74% - 80% among HCWs [11,16]. Ideally, HCWs are expected to have adequate knowledge on how masks can prevent the spread of infectious droplets thus more likely to wear appropriate face coverings. As demonstrated in previous studies, mask use increased in high risk situations, such as contact with a patient with febrile respiratory illness and the presence of medical conditions in the HCWs [30,31]. In this study, the compliance in health facilities (HFs) was higher than other sampled locations, possibly due to perceived risk of infections in HFs. Further, health authorities are known to be designers of public health measures, it could be expected that individuals accessing the health services irrespective of their health conditions, are likely to adhere to prevention guidelines for the fear of being denied access or attend to.

In this study we found poor face mask compliance among persons using public transport i.e. mini or coach buses. Studies indicate benefits of correct face masks usage to prevent the transmission of COVID-19 infection caused by public transportation exposure [13,32]. A case study conducted on one patient who did not wear a face mask in the first vehicle while another wore a face mask in the second vehicle, during COVID-19 epidemic from Chongqing, China, showed that many passengers who did not wear face masks on the same coach bus developed respiratory symptoms while all passengers on the second vehicle did not and qRT-PCR test results were negative [1,13,16]. Adherence to compulsory face masks use could be significantly improved through restrictive measures by authorities on public transit. Our study recommends reinforcement of these orders on the general population through random inspections and monitoring of public transport, and sensitising commuters on the importance of protecting themselves and others against COVID-19.

Our study revealed that participants at learning institutions were twice more likely to wear face masks correctly than those observed at the bus stations. This compares to a study that involved 30383 students from 62 countries that assessed the impact of the COVID-19 pandemic on life of higher education students globally [33,34]. The study noted that despite deficient computer skills and the perception of a higher workload among learners, the majority of students were satisfied with the support provided by teaching staff and their universities´ public relations hence willing to adapt the new teaching environment [35]. Students could be mainly concerned with their future professional career and studies hence willing to adapt particular hygienic behaviours such as wearing masks, washing hands, staying at home and maintaining social distancing. Moreover, students may be influenced to adopt such habits especially when feel are accepted and valued by other friends. Further, learners and teachers alike, could have more knowledge on the COVID-19 pandemic preventive measures, mode of transmission as well as susceptible groups for the coronavirus infection. A study among Jordanian medical students showed encouraging response to the COVID-19 pandemic where more than 80% of study participants adopted social isolation strategies, regular hand washing, and enhanced personal hygiene measures as their first line of defence against the virus [34,36]. High level of knowledge on COVID-19 could likely enhance the implementation proper strategies to prevent its spread.

Our study reports an exceptionally low (2%) face masks usage among persons observed at markets. This is a serious public health concern because markets are usually crowded spaces and likely to exacerbate the spread the SARS-CoV-2 virus. If markets and shopping mall goers adjusted to universal face-mask use, the potential benefits of people wearing face coverings could be greatly realised. Reports indicate that unlike SARS-CoV-1 and MERS-CoV, the SARS-CoV-2 virus is likely to infect people during incubation, and asymptomatic patients also have potential infectivity [2,4,10,12]. It is therefore important that everyone carry a face mask when they leave home in order to tackle coronavirus. Our findings are subject to a few limitations. The use of convenient sampling procedure could limit the study´s representativeness of the face-masks uptake among the Zambians. The study was conducted in two provincial towns (classified as urban and sub-urban), thereby limiting the generalizability to general populations because rural areas and persons who may have no access to social services such as shopping malls, schools, markets and bus stations, may hold different views and options on the subject. Furthermore, the study was conducted at one point in time, which was at the onset of the second wave of COVID-19 in Zambia following approximately three months of relatively low case counts nationwide; thus, the public´s risk perception might have been low during this time and a similar study done during the peak of the second wave might have resulted in substantially different findings. Additionally, there could age misclassification because observations were sampled without recording information on the persons observed (i.e. cut-off between teenage and adult was based on stature or appearance). Therefore, we recommend for further studies to understand the prevalence and factors associated with face masks utilization across age, gender and location (urban vs rural) in the country.

## Conclusion

Although face masks are one of NPIs that aim to prevent SARS-CoV-2 transmission in the community, there was poor face masks usage probably due to low-risk perception about COVID-19 pandemic among Mansa and Lusaka residents. This could have been related to when the study was conducted. Among those observed wearing face masks, about half had masked-up correctly; being a market, school, and female were associated with wearing face-masks correctly. We found poor face mask compliance among persons using public transport posing a challenge in preventing the spread of COVID-19 in an environment experiencing community transmission. The Ministry of Health should ensure that wearing of masks is understood and correctly applied by the population, and that masks are used in combination with hand hygiene and knowledge of proper use and disposal. There need for extra public health messaging to dispel misconceptions about COVID-19 among residents. We recommend for further restrictive measures by authorities in public spaces including random inspections of public transport and enhanced compliance checks in shopping malls, markets and schools. Follow up studies to understand reasons for poor face masks adherence across age, gender and location should be considered to appropriately inform policy.

### What is known about this topic

- Face masks use is a basic non-pharmaceutical intervention recommended for prevention of respiratory infectious diseases;
- Appropriate use and disposal of face masks could reduce the risk of infection in the absence of a safe and effective vaccine to protect those at risk of severe COVID-19;
- Wearing a face mask or cloth face covering in public settings is mandatory in some countries to control the spread of COVID-19.

### What this study adds

- Wearing a face mask or cloth face covering in public settings is mandatory in some countries to control the spread of COVID-19;
- Wearing a face mask or cloth face covering in public settings is mandatory in some countries to control the spread of COVID-19;
- High levels of incorrect face masks use probably due to: inadequate sensitization on proper use and disposal of masks, low-risk perception about COVID-19 pandemic and relaxed restrictive measures by authorities in public spaces.
